# Sleep disorders among elderly hypertensive patients: associated factors and implications for management

**DOI:** 10.3389/fcvm.2026.1769940

**Published:** 2026-07-06

**Authors:** Cheng Hong, Jiaying Gu, Xiaohua Cheng, Xuechun Jiang, Yun Wang, Lijuan Zhao

**Affiliations:** Department of Cardiovascular Medicine, Taicang TCM Hospital Affiliated to Nanjing University of Chinese Medicine, Suzhou, Jiangsu Province, China

**Keywords:** associated factors, care, elderly, hypertension, sleep disorders, strategies

## Abstract

**Background:**

Sleep disorders are common comorbidities in elderly hypertensive patients, forming a vicious cycle with hypertension that exacerbates disease progression and impairs quality of life. This study aimed to explore the factors independently associated with sleep disorders in elderly hypertensive patients and provide evidence for clinical management.

**Methods:**

A cross-sectional survey was conducted among elderly hypertensive patients admitted to our hospital from June 2025 to October 2025. Data were collected using a self-designed, psychometrically validated questionnaire. Univariate analysis, correlation analysis, and multivariate logistic regression were used to identify associated factors of sleep disorders.

**Results:**

The prevalence of sleep disorders in the enrolled population was 39.02% (206/528). Univariate and correlation analyses showed significant differences and correlations between sleep disorders and age, body mass index (BMI), education level, family per capita monthly income, hypertension course, hypertension grade, regular exercise, and pre-bedtime electronic device use (all *P* < 0.05). Multivariate logistic regression analysis revealed that advanced age (≥70 years), prolonged hypertension course (≥10 years), high hypertension grade (grade 2–3), lack of regular exercise, and frequent pre-bedtime electronic device use (≥3 times/week) were independently associated with sleep disorders (all *P* < 0.001). The regression model exhibited good fit (Hosmer–Lemeshow test, *P* = 0.352) and showed moderate discriminative ability in exploratory analysis.

**Conclusion:**

Sleep disorders are highly prevalent among elderly hypertensive patients. Advanced age, prolonged hypertension duration, severe hypertension, sedentary lifestyle, and frequent pre-bedtime electronic device use were identified as factors independently associated with sleep disturbances. Clinical practice should adopt integrated management strategies, including individualized blood pressure control, routine sleep assessment for high-risk groups, targeted lifestyle interventions, and multidisciplinary collaboration.

## Introduction

Against the backdrop of the global aging population, elderly hypertension has become a major challenge in the field of public health worldwide (1). Its prevalence among individuals aged 60 years and above exceeds 50%, and it is often accompanied by degenerative changes in multiple organ functions and an extensive spectrum of complications (2, 3). As one of the most common comorbidities in elderly hypertensive patients, sleep disorders have a clinical incidence as high as 35%∼60%, which is significantly higher than the 15%∼20% in the general elderly population. This wide range has been reported in multiple epidemiological surveys specific to hypertensive older adults (4, 5). They are mainly characterized by difficulty falling asleep, increased nighttime awakenings, early morning awakening, fragmented sleep structure, and daytime sleepiness (6). This high incidence is not accidental, as there is a clear pathophysiological association between the two: hypertension can directly disrupt sleep rhythms by elevating nighttime blood pressure, impairing cerebral blood flow autoregulation, and—particularly in poorly controlled or severe hypertension—causing discomfort such as headache and dizziness; conversely, long-term insufficient sleep or poor sleep quality can further activate the sympathetic nervous system, increase the activity of the renin-angiotensin-aldosterone system, and lead to disorders of the circadian rhythm of blood pressure, forming a “hypertension–sleep disorder” vicious cycle (7–9). This cycle not only reduces patients' quality of life but also significantly increases the risk of cardiovascular and cerebrovascular events (such as stroke and myocardial infarction), cognitive decline, and mental health problems such as anxiety and depression, posing great challenges to clinical management (10).

Despite the crucial impact of sleep disorders on the disease control and prognosis of elderly hypertensive patients, there remains a significant gap in attention in current clinical practice: the management focus of most medical institutions is concentrated on controlling blood pressure values, lacking systematic norms for the screening, assessment, and intervention of sleep status (11); some clinicians have insufficient understanding of the interaction mechanism between the two, and often attribute sleep problems to a natural phenomenon of aging, failing to take timely targeted measures (12). In addition, sleep disorders in these patients are influenced by a multitude of intertwined variables—such as demographic characteristics, disease severity (e.g., grade, duration), lifestyle habits (physical activity, screen time), and psychological distress—yet existing studies have rarely integrated these domains into a single analytic framework (13, 14). Moreover, no unified risk assessment system or intervention guidelines have been established. Therefore, systematically revealing the core associated factors of concurrent sleep disorders in elderly hypertensive patients and clarifying the strength of action and correlation of each factor are of great practical significance for filling the gap in clinical cognition and optimizing management strategies.

Based on the above clinical status and research gaps, this study aims to clarify the epidemiological characteristics of sleep disorders in elderly hypertensive patients, systematically identify their key associated factors through a cross-sectional survey, and discuss implications for prevention and management. It is expected to provide high-quality evidence for clinical individualized sleep management, thereby contributing to breaking the “hypertension–sleep disorder” vicious cycle, and improving patient prognosis.

## Methods

### Ethical approval

This study protocol was reviewed and approved by the Medical Ethics Committee of Taicang Hospital of Traditional Chinese Medicine (Ethical Approval No.: 2024044), and the entire research process was conducted in strict accordance with the *Declaration of Helsinki* (15). Prior to the initiation of the study, uniformly trained investigators explained the research purpose, technical route, scope of data collection, data usage methods, and confidentiality measures to each potential participant or their legal representative in plain language. It was explicitly stated that participation was voluntary, and participants could withdraw from the study at any stage without providing a reason, with no impact on their routine diagnosis and treatment services and rights. All participants signed a written informed consent form after being fully informed of the study details.

### Study design

A cross-sectional survey design was adopted in this study, focusing on the factors associated with sleep disorders in elderly hypertensive patients. Elderly hypertensive patients attending the outpatient department of Cardiology in our hospital were selected as research subjects via convenience sampling, with the survey period spanning from June 2025 to October 2025. In this study, “presence or absence of sleep disorders” was set as the outcome variable, while sociodemographic characteristics (gender, age, income, etc.), disease-related indicators (course of hypertension, hypertension grade, BMI, blood pressure values, antihypertensive medication classes), lifestyle factors (regular exercise, electronic device use before bedtime, etc.), and environmental and psychological factors were defined as independent variables. Data were collected through standardized questionnaires, and factors independently associated with sleep disorders were systematically identified by combining univariate analysis, correlation analysis, and multivariate regression analysis.

### Sample size calculation

Multivariate logistic regression analysis was employed as the primary statistical method in this study, and the sample size was estimated for the precision of prevalence, using the formula:

*n* = (Z₁₋*α*/₂)² × P(1 – P)/d². Where Z₁₋*α*/₂ = 1.96 (*α*=0.05, two-tailed), *P* = 0.39 (anticipated prevalence based on pre-survey), and allowable error d = 0.05. The power concept does not apply to this precision-based estimation; the approach aimed to ensure a 95% confidence interval width of ±5% around the observed prevalence. Substituting these values into the formula yielded a minimum required sample size of 385 cases.

Considering potential questionnaire recovery rates, invalid questionnaire exclusion rates (estimated at 10%–15%) in actual surveys, and the number of independent variables in multivariate analysis (14 core independent variables in this study), the sample size was further expanded in accordance with the criterion of “15–20 events per variable” for logistic regression (16), with a final planned sample size of 550 cases. In the actual survey, a total of 528 valid participants were enrolled, which met the sample size requirements for multivariate logistic regression analysis (valid sample size ≥ minimum sample size and ≥ 10 times the number of independent variables).

### Study population

Eligible participants were elderly individuals aged ≥ 60 years who met the diagnostic criteria for hypertension as specified in the “Chinese Guidelines for the Management of Hypertension ”^17^ (i.e., systolic blood pressure ≥ 140 mmHg and/or diastolic blood pressure ≥ 90 mmHg measured on three non-consecutive days) and had a disease course of at least 3 months. All participants possessed clear consciousness, normal cognitive function (defined as a score ≥ 24 on the Mini-Mental State Examination, MMSE), and the ability to communicate effectively with basic reading comprehension skills, enabling them to complete the questionnaire independently or with the assistance of investigators. Additionally, participants voluntarily agreed to participate in the study by signing an informed consent form and had no history of major diseases or surgical procedures within the recent month, with relatively stable clinical conditions. Individuals were excluded from the study if they had concurrent severe organ failure involving the heart, liver, kidneys, or other vital organs (e.g., New York Heart Association Class Ⅳ heart failure, decompensated cirrhosis, uremia) or suffered from mental illnesses (e.g., schizophrenia, severe anxiety and depression), severe cognitive dysfunction (MMSE score < 24), or neurological diseases (e.g., Parkinson's disease, severe sequelae of stroke) that could interfere with sleep assessment. Also excluded were those who had experienced acute cardio-cerebrovascular events (e.g., cerebral hemorrhage, cerebral infarction, myocardial infarction), severe infections (e.g., sepsis), or major surgeries within 1 month prior to enrollment, as well as patients with long-term use of sedative-hypnotic drugs (≥ 5 times per week) or other medications potentially affecting sleep (e.g., high-dose glucocorticoids). Participants currently involved in other similar studies or those unable to complete the survey due to severe hearing or visual impairment were also excluded.

### Data collection

A self-designed questionnaire titled *Sleep Disorder Assessment Questionnaire for Elderly Hypertensive Patients* was developed as the data collection instrument, adhering to a rigorous standardized process encompassing literature review, conceptual framework construction, item formulation, expert validation, pre-survey revision, and reliability and validity testing. The full instrument is provided as Supplementary Material. Initially, a systematic literature search was conducted across databases including PubMed, CNKI, and Wanfang Data to synthesize core variables and evaluation indicators from existing studies on hypertension and sleep disorders in the elderly population, thereby establishing the preliminary structural framework of the questionnaire. Subsequently, four key dimensions—sociodemographic and baseline characteristics, hypertension-related clinical indicators, core sleep status parameters, and potential sleep-influencing factors—were integrated into the questionnaire design, with specific items formulated to comprehensively capture the hypothesized associated factors relevant to the research objectives.

For expert validation, five interdisciplinary specialists (three Chief Physicians in Cardiology, one Associate Chief Physician in Geriatrics, and one Attending Physician specializing in Sleep Medicine) were invited to conduct two rounds of Delphi consultations. The expert authority coefficient (Cr) was 0.87, indicating high credibility of the expert panel. Feedback focused on the clarity of item expression, logical coherence of the questionnaire structure, and relevance of content to the research focus, leading to the deletion of 2 redundant items and revision of 3 ambiguously phrased items to enhance comprehensibility and accuracy. A pre-survey was then administered to 30 eligible elderly hypertensive patients (who were subsequently excluded from the final sample; their demographic and clinical profiles were comparable to those of the main cohort) to assess item performance, with item difficulty coefficients ranging from 0.32 to 0.78 and item discrimination coefficients between 0.25 and 0.63—values within the acceptable range for psychometric instruments—prompting further refinement of item wording and structure.

The final version of the questionnaire comprises three distinct sections. The first section collects sociodemographic and hypertension-related descriptive information (including systolic/diastolic blood pressure and antihypertensive medication classes), without generating a summative score. It includes 13 items covering gender, age, marital status, education level, place of residence, family per capita monthly income, body mass index (BMI), duration of hypertension, hypertension grade, antihypertensive medication regimen details, smoking and drinking history, and regular exercise habits. The second section focuses on core sleep status assessment (10 items), covering bedtime, actual sleep duration, sleep onset latency, nighttime awakenings, the impact of nocturia on sleep, and other sleep-related parameters. Items 3–9 in this section utilize a 4-point Likert scale (0 = none, 1 = <1 time/week, 2 = 1–2 times/week, 3 = ≥3 times/week), with a total score ≥8 indicating the presence of significant sleep disorders. This cut-off was established through two consecutive phases: expert consensus during the Delphi consultation recommended an initial threshold that best discriminated clinically meaningful sleep complaints, and pilot testing (*n* = 30) against a structured sleep interview confirmed a sensitivity of 0.85 and specificity of 0.79 at this score, supporting its diagnostic utility. The third section includes supplementary items (4 items) investigating potential sleep-influencing factors, such as the quietness of the living environment, frequency of electronic device use before bedtime, pre-sleep dietary habits, and emotional state.

Psychometric testing confirmed the reliability and validity of the questionnaire. The overall Cronbach's *α* coefficient was 0.826, with Cronbach's α coefficients ranging from 0.763 to 0.851 across the three sections, demonstrating good internal consistency. Test-retest reliability, assessed by re-administering the questionnaire to 30 patients after a 2-week interval, yielded an intraclass correlation coefficient (ICC) of 0.842, indicating stable measurement results over time. Content validity was evaluated using the content validity index (CVI), with an overall CVI of 0.89 and item-level content validity indices (I-CVI) ranging from 0.83 to 0.95, confirming strong alignment between questionnaire content and the research objectives. Exploratory factor analysis was conducted on the full study sample (*n* = 528) and extracted three common factors, accounting for 68.35% of the total variance, with all item factor loadings exceeding 0.60, further verifying the structural validity of the instrument.

### Statistical analysis

Data processing and analysis were performed using SPSS 26.0 statistical software. Normality of measurement data was first tested using the Shapiro–Wilk test. Data conforming to a normal distribution were expressed as mean ± standard deviation, and inter-group comparisons were conducted using independent samples t-test. Data not conforming to a normal distribution were expressed as median (interquartile range) [M (Q1, Q3)], and inter-group comparisons were performed using the Wilcoxon rank-sum test. Count data were expressed as number (percentage) [n (%)], with inter-group comparisons using the chi-square test; for ordinal categorical data, the rank-sum test was used. Pearson correlation analysis (for continuous variables) and Spearman rank correlation analysis (for categorical variables) were employed to explore the correlations between various factors and sleep disorders. Variables with *P* < 0.05 in univariate analysis were included in multivariate binary logistic regression analysis (using the Enter method) to identify factors independently associated with sleep disorders in elderly hypertensive patients. The discriminative ability of the model combining these associated factors was explored using receiver operating characteristic (ROC) curve analysis as a secondary analysis; detailed results are provided in Supplementary Figure S1 and Supplementary Table S1. A two-tailed test was used, and a *P*-value <0.05 was considered statistically significant.

## Results

A total of 528 elderly hypertensive patients were enrolled in this study, including 206 patients with sleep disorders (prevalence rate: 39.02%) and 322 patients without sleep disorders. As shown in Table 1, univariate analysis revealed significant differences in age, body mass index (BMI), education level, family per capita monthly income, hypertension course, hypertension grade, regular exercise, and electronic device use before bedtime between the two groups (all *P* < 0.05). Systolic and diastolic blood pressure measurements, as well as the distribution of antihypertensive medication classes, were comparable between groups (Table 1). Specifically, patients with sleep disorders were older, had a higher BMI, longer hypertension course, and higher proportion of grade 2–3 hypertension compared with those without sleep disorders. Additionally, the proportion of patients with primary school education or below, family per capita monthly income <5000 yuan, no regular exercise, and frequent electronic device use before bedtime (≥3 times/week) was significantly higher in the sleep disorder group. In contrast, no significant differences were observed in gender, marital status, residence, regular antihypertensive medication adherence, smoking history, or drinking history between the two groups (all *P* > 0.05).

**Table 1:** Baseline characteristics of participants and univariate analysis of sleep disorders (*n* = 528).

Variables	Sleep disorder group (*n* = 206)	No sleep disorder group (*n* = 322)	t/*χ*²	P
Age (y)	73.68 ± 5.24	67.35 ± 6.81	10.256	<0.001
BMI (kg/m²)	25.86 ± 3.12	24.15 ± 2.87	5.328	0.002
Systolic blood pressure (mmHg)	146.3 ± 14.2	144.5 ± 13.8	1.46	0.145
Diastolic blood pressure (mmHg)	85.2 ± 9.6	84.1 ± 10.2	1.23	0.219
Gender (Male/Female)	108/98	175/147	0.186	0.666
Marital status (Married/Other)	156 (75.73%)	263 (81.68%)	2.954	0.086
Education level (Primary school and below/Above primary school)	132 (64.08%)	168 (52.17%)	7.892	0.005
Residence (Urban/Rural/Township)	85/72/49	158/105/59	3.215	0.201
Family per capita monthly income (<5,000 yuan/≥5,000 yuan)	130 (63.11%)	170 (52.80%)	6.358	0.012
Hypertension course (y)	11.35 ± 4.26	7.28 ± 3.95	11.582	<0.001
Hypertension grade (Grade 2–3/Grade 1)	162 (78.64%)	185 (57.45%)	32.691	<0.001
Antihypertensive medication class				
ACEI/ARB	89 (43.20%)	148 (45.96%)	0.586	0.444
CCB	95 (46.12%)	137 (42.55%)	0.789	0.374
Diuretic	38 (18.45%)	55 (17.08%)	0.243	0.622
*β*-blocker	42 (20.39%)	60 (18.63%)	0.362	0.547
Regular antihypertensive medication (Yes/No)	178 (86.41%)	289 (89.75%)	1.568	0.211
Smoking (Yes/No/Quit)	65/112/29	98/185/39	2.135	0.344
Drinking (Yes/No/Quit)	58/125/23	89/196/37	1.872	0.392
Regular exercise (Yes/No)	42 (20.39%)	138 (42.86%)	35.782	<0.001
Electronic device use before bedtime (≥3 times/week/<3 times/week)	128 (62.14%)	115 (35.71%)	48.963	<0.001

BMI, body mass index; ACEI, angiotensin-converting enzyme inhibitor; ARB, angiotensin receptor blocker; CCB, calcium channel blocker. *P*-values derived from chi-square test for categorical variables and independent samples t-test for continuous variables. All comparisons are two-tailed.

Correlation analysis results (Table 2) indicated that age, BMI, education level, family per capita monthly income, hypertension course, hypertension grade, regular exercise, and electronic device use before bedtime were significantly correlated with the occurrence of sleep disorders (all *P* < 0.05). Age (r = 0.428), hypertension course (r = 0.465), and hypertension grade (r = 0.512) showed moderate positive correlations with sleep disorders, while regular exercise (r = −0.325) exhibited a moderate negative correlation. BMI (r = 0.186), education level (r = 0.158), family per capita monthly income (r = −0.136), and electronic device use before bedtime (r = 0.386) showed weak to moderate correlations with sleep disorders. No significant correlations were found between gender, marital status, residence, regular antihypertensive medication adherence, smoking history, drinking history, and sleep disorders (all *P* > 0.05).

**Table 2:** Correlation analysis between sleep disorders and influencing factors in elderly hypertensive patients.

Variables	r	P
Age (y)	0.428	<0.001
BMI (kg/m²)	0.186	0.002
Gender	0.021	0.666
Marital status	−0.075	0.086
Education level	0.158	0.005
Residence	0.082	0.201
Family per capita monthly income	−0.136	0.012
Hypertension course (y)	0.465	<0.001
Hypertension grade	0.512	<0.001
Regular antihypertensive medication	−0.048	0.211
Smoking history	0.052	0.344
Drinking history	0.046	0.392
Regular exercise	−0.325	<0.001
Electronic device use before bedtime	0.386	<0.001

*P*-values from Pearson correlation for continuous variables and Spearman rank correlation for categorical/ordinal variables. All tests two-tailed.

To identify factors independently associated with sleep disorders in elderly hypertensive patients, variables with *P* < 0.05 in univariate analysis were included in multivariate logistic regression analysis. The variable assignment is shown in Table 3. Multivariate logistic regression analysis (Table 4) showed that age ≥70 y, hypertension course ≥10 y, hypertension grade 2–3, no regular exercise, and electronic device use before bedtime ≥3 times/week were independently associated with sleep disorders (all *P* < 0.001). Specifically, patients aged ≥70 y had an odds ratio (OR) of 2.376 (95%CI: 1.685–3.352) compared with those aged <70 y. Patients with hypertension course ≥10 y had an OR of 2.531 (95%CI: 1.806–3.558), and those with grade 2–3 hypertension had an OR of 2.875 (95%CI: 2.042–4.046) relative to grade 1. The OR for no regular exercise was 2.186 (95%CI: 1.563–3.058), and for frequent electronic device use before bedtime (≥3 times/week) was 2.618 (95%CI: 1.895–3.612). The regression model passed the Hosmer-Lemeshow goodness-of-fit test (*P* = 0.352), indicating a good model fit. Exploratory ROC analysis of the combined five factors (Supplementary Figure S1) yielded moderate discriminative ability, with detailed sensitivity and specificity values across scoring thresholds presented in Supplementary Table S1.

**Table 3:** Variable assignment for multivariate logistic regression analysis of associated factors.

Factors	Variables	Assignment
Sleep disorder	Dependent variable	Yes=1, No = 0
Age	Independent variable	≥70 y = 1, <70 y = 0
Hypertension course	Independent variable	≥10 y = 1, <10 y = 0
Hypertension grade	Independent variable	Grade 2–3 = 1, Grade 1 = 0
Regular exercise	Independent variable	No = 1, Yes=0
Electronic device use before bedtime	Independent variable	≥3 times/week=1, <3 times/week=0

**Table 4:** Multivariate logistic regression analysis of factors associated with sleep disorders in elderly hypertensive patients.

Variables	*β*	Wald	OR	95%CI	P
Age ≥70 y	0.865	28.352	2.376	1.685–3.352	<0.001
Hypertension course ≥10 y	0.928	32.691	2.531	1.806–3.558	<0.001
Hypertension grade 2–3	1.056	41.285	2.875	2.042–4.046	<0.001
No regular exercise	0.782	25.147	2.186	1.563–3.058	<0.001
Electronic device use ≥3 times/week	0.963	38.752	2.618	1.895–3.612	<0.001

The supplementary ROC analysis based on these five factors is presented in Supplementary Figure S1 and Supplementary Table S1.

## Discussion

This study explored the factors associated with sleep disorders among elderly hypertensive patients, with a detected prevalence of 39.02% in the enrolled 528 participants. This prevalence is consistent with the range of 35%–60% reported in most epidemiological studies (6, 18, 19) focusing on this population, confirming that sleep disorders are a highly prevalent comorbidity in elderly hypertensive individuals. The findings identified five factors independently associated with sleep disorders in this cohort, shedding light on the complex interplay between demographic characteristics, disease severity, lifestyle behaviors, and sleep health, and providing empirical evidence that aligns with and extends the conclusions of existing research (20, 21).

Age stands out as a prominent associated factor, reflecting the cumulative impact of physiological aging on sleep regulation. With advancing age, the body's circadian rhythm system undergoes natural declines, including reduced melatonin secretion and altered sleep architecture characterized by shortened slow-wave sleep and increased sleep fragmentation (22, 23). This is consistent with the findings of a large-scale cross-sectional study involving elderly hypertensive patients, which also identified advanced age as a key predictor of sleep disturbance risk (24). Additionally, older individuals often experience more pronounced vascular aging and related comorbidities, which may further disrupt cerebral blood flow stability and exacerbate nocturnal discomfort, thereby interfering with sleep continuity (25). This highlights the need for prioritized sleep assessment and proactive intervention in elderly hypertensive patients, particularly those in the advanced age group.

The duration and severity of hypertension are closely linked to sleep disorder risk, underscoring the critical role of disease control in preserving sleep health. Long-term hypertension can induce structural and functional changes in cerebral vasculature, impairing autoregulatory capacity and leading to inadequate cerebral perfusion during sleep, as documented in both hypertensive and sleep research (26, 27). These consistent observations across studies (28, 29) emphasize the importance of not only achieving target blood pressure levels but also optimizing blood pressure rhythm through individualized management strategies.

Lack of regular exercise emerges as a modifiable associated factor, highlighting the significance of lifestyle interventions in sleep health promotion. Physical activity is known to regulate circadian rhythms, reduce sympathetic activation, and alleviate psychological stress, all of which contribute to improved sleep quality (19). Conversely, sedentary behavior among elderly hypertensive patients may lead to reduced physical fitness, metabolic imbalance, and increased psychological distress, creating a favorable environment for sleep disturbances (30). It also underscores the potential value of integrating structured exercise programs into routine management for this patient population (31).

Frequent electronic device use before bedtime represents a modern lifestyle factor with substantial impact on sleep health. Electronic screens emit blue light, which suppresses melatonin secretion and disrupts circadian rhythm alignment, while prolonged engagement with electronic devices can increase cognitive arousal and mental stimulation, delaying sleep onset and reducing sleep depth (32). For elderly hypertensive patients, whose circadian regulation is already compromised, these effects may be amplified. This is supported by a recent study (33) focusing on geriatric populations, which revealed that pre-bedtime electronic device use was associated with a higher risk of sleep onset insomnia, a result consistent with the odds ratio identified in the present analysis. This highlights the need to prioritize behavioral interventions targeting screen time reduction in clinical sleep health education for elderly hypertensive patients.

Based on the identified associated factors and their alignment with evidence from related studies, a comprehensive approach to clinical management, nursing, and treatment is recommended to address sleep disorders in elderly hypertensive patients. Individualized blood pressure management should prioritize long-acting antihypertensive agents that promote stable nocturnal blood pressure, with regular 24-hour blood pressure monitoring to guide treatment adjustments and achieve a physiological dipper rhythm (34). Routine sleep assessment using standardized tools should be integrated into clinical practice, particularly for high-risk groups such as older patients, those with long-standing or severe hypertension, and individuals with sedentary lifestyles or frequent pre-bedtime electronic device use (35). Sleep health education should be tailored to address modifiable risk factors, including guidance on maintaining consistent sleep schedules, optimizing the sleep environment to reduce noise and light exposure, and adopting relaxing pre-sleep routines that avoid electronic device use. Non-pharmacological interventions such as sleep hygiene training, relaxation techniques, and cognitive-behavioral strategies should be implemented for patients with established sleep disorders (36). Promotion of healthy lifestyles should include personalized exercise recommendations aligned with individual physical capabilities, as endorsed by current hypertension and lifestyle guidelines (37–39), ranging from brisk walking and tai chi to chair-based exercises for those with mobility limitations, alongside dietary guidance to avoid sleep-disrupting substances before bedtime (40). Multidisciplinary collaboration among cardiologists, geriatricians, nurses, and allied health professionals is essential to deliver integrated care that addresses both hypertension management and sleep health, with regular follow-up to monitor progress and adjust interventions as needed (41).

This study has several limitations that should be considered when interpreting the results. The cross-sectional design precludes the establishment of causal relationships between the identified factors and sleep disorders, and all associations reported should be interpreted accordingly. The use of convenience sampling from a single hospital's cardiology outpatient department may introduce selection bias, limiting the generalizability of findings to the broader elderly hypertensive population, especially those managed in community settings or with milder disease severity. Sleep disorders were assessed using a self-reported questionnaire rather than objective measures such as polysomnography, which may lead to subjective reporting biases and incomplete characterization of sleep disturbances such as sleep-disordered breathing. Potential confounding factors, including mental health conditions such as anxiety and depression, chronic pain, and other comorbidities, were not fully evaluated, which may have influenced the observed associations. Additionally, the study did not explore the underlying biological mechanisms linking the identified associated factors to sleep disorders, leaving room for further investigation into pathophysiological pathways such as neurohumoral activation and inflammatory responses. Despite these limitations, the findings provide valuable insights into the factor profile of sleep disorders in elderly hypertensive patients and offer practical guidance for clinical practice, highlighting the importance of integrated management that addresses both disease control and sleep health.

## Conclusion

In conclusion, the present study finds that sleep disorders are highly prevalent among elderly hypertensive patients. Advanced age, prolonged hypertension course, high hypertension grade, lack of regular exercise, and frequent electronic device use before bedtime were independently associated with sleep disturbances in this cross-sectional sample. These findings clarify the key contributors to sleep health impairment in elderly hypertensive individuals and highlight the bidirectional interplay between hypertension-related pathological changes and lifestyle factors in disrupting sleep regulation. To address these issues, clinical practice should adopt integrated management strategies that combine individualized blood pressure control to optimize nocturnal blood pressure rhythms, routine sleep assessment for high-risk groups, targeted lifestyle interventions including the promotion of regular physical activity and restriction of pre-bedtime electronic device use, and multidisciplinary collaboration to deliver comprehensive care for both hypertension and sleep disorders. Future research should employ longitudinal study designs to verify the causal nature of these relationships and incorporate objective sleep monitoring techniques such as polysomnography to enhance the accuracy of sleep disorder diagnosis, and explore the underlying molecular and neurophysiological mechanisms; additionally, randomized controlled trials are warranted to evaluate the efficacy of tailored intervention programs in improving sleep quality and long-term prognosis for elderly hypertensive patients with sleep disorders.

## Data Availability

The raw data supporting the conclusions of this article will be made available by the authors, without undue reservation.

## Supplementary material

Supplementary Figure S1 Receiver operating characteristic (ROC) curve for the set of associated factors in elderly hypertensive patients (exploratory analysis). Detailed results are in Supplementary Table S1.
