# Supplementary figures and images for: Sleep disorders among elderly hypertensive patients: associated factors and implications for management
Source: Front Cardiovasc Med. 2026 Jul 6;13:1769940. doi: 10.3389/fcvm.2026.1769940 (PMC13381476; doi:10.3389/fcvm.2026.1769940)

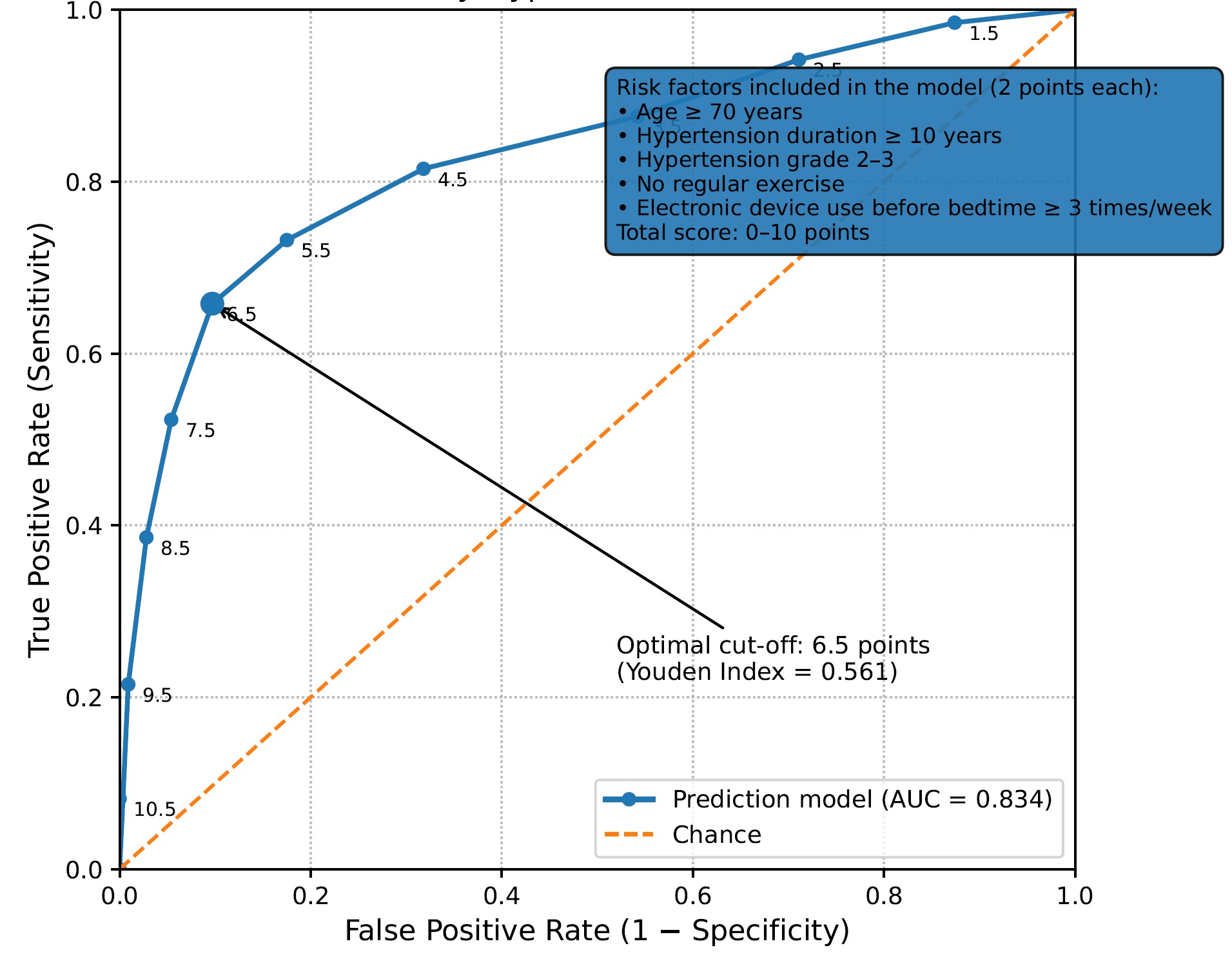

Supplement: Supplementary Figure S1 — Receiver operating characteristic (ROC) curve for the set of associated factors in elderly hypertensive patients (exploratory analysis). Detailed results are in Supplementary Table S1. [file Image1.jpeg]
