# Supplementary material for: Sleep disorders among elderly hypertensive patients: associated factors and implications for management
Source: Front Cardiovasc Med. 2026 Jul 6;13:1769940. doi: 10.3389/fcvm.2026.1769940 (PMC13381476; doi:10.3389/fcvm.2026.1769940)
Supplement: Supplementary file 2 [file Table1.docx]

Table S1 Sensitivity and specificity of the prediction model for sleep disorders in elderly hypertensive patients

| Total score​ | Sensitivity​ | Specificity​ | Youden Index​ |
| --- | --- | --- | --- |
| 0.5​ | 1.000​ | 0.000​ | 0.000​ |
| 1.5​ | 0.985​ | 0.126​ | 0.111​ |
| 2.5​ | 0.942​ | 0.289​ | 0.231​ |
| 3.5​ | 0.876​ | 0.458​ | 0.334​ |
| 4.5​ | 0.815​ | 0.682​ | 0.497​ |
| 5.5​ | 0.732​ | 0.825​ | 0.557​ |
| 6.5​ | 0.658​ | 0.903​ | 0.561​ |
| 7.5​ | 0.523​ | 0.946​ | 0.469​ |
| 8.5​ | 0.386​ | 0.972​ | 0.358​ |
| 9.5​ | 0.215​ | 0.991​ | 0.206​ |
| 10.5​ | 0.082​ | 1.000​ | 0.082​ |
| 11.0​ | 0.000​ | 1.000​ | 0.000​ |

​Note: The scoring criteria of the prediction model refer to the 5 risk factors included in Table 4, with 2 points assigned to each risk factor. The total score ranges from 0 to 10 points (Age ≥70 y=2 points, Hypertension course ≥10 y=2 points, Hypertension grade 2-3=2 points, No regular exercise=2 points, Electronic device use ≥3 times/week=2 points). The optimal cut-off value is 6.5 points (Youden Index=0.561).​
