# Supplementary material for: Sleep disorders among elderly hypertensive patients: associated factors and implications for management
Source: Front Cardiovasc Med. 2026 Jul 6;13:1769940. doi: 10.3389/fcvm.2026.1769940 (PMC13381476; doi:10.3389/fcvm.2026.1769940)
Supplement: Supplementary file 3 [file Datasheet1.docx]

**Sleep Disorders Among Elderly Hypertensive Patients Questionnaire**

**Dear Patient:**

This questionnaire is designed to assess sleep status and related influencing factors in elderly hypertensive patients. All information will be used exclusively for medical research and will be kept strictly confidential. There are no right or wrong answers. Please complete the questionnaire based on your actual situation over the past month. Thank you for your cooperation.

**Part I: Baseline Information and Hypertension-Related Factors**

| **No.** | **Question** | **Options** |
| --- | --- | --- |
| 1 | Gender | A. Male B. Female |
| 2 | Age | A. 60–65 years B. 66–70 years C. 71–75 years D. ≥76 years |
| 3 | Marital status | A. Married B. Divorced C. Widowed D. Unmarried |
| 4 | Education level | A. Primary school or below B. Junior high school C. Senior high school / technical secondary school D. College or above |
| 5 | Place of residence | A. Urban B. Rural C. Township |
| 6 | Family per capita monthly income (CNY) | A. <3,000 B. 3,000–4,999 C. 5,000–9,999 D. ≥10,000 |
| 7 | Body mass index (BMI, kg/m²) (calculated as weight [kg] / height [m]²; may be assisted by the investigator) | A. <18.5 (underweight) B. 18.5–23.9 (normal) C. 24.0–27.9 (overweight) D. ≥28.0 (obese) |
| 8 | Duration of hypertension | A. <1 year B. 1–5 years C. 6–10 years D. >10 years |
| 9 | Hypertension grade | A. Grade 1 (systolic 140–159 mmHg and/or diastolic 90–99 mmHg) B. Grade 2 (systolic 160–179 mmHg and/or diastolic 100–109 mmHg) C. Grade 3 (systolic ≥180 mmHg and/or diastolic ≥110 mmHg) D. Unknown |
| 10 | Regular use of antihypertensive medication | A. Yes (taken daily as prescribed) B. No (taken occasionally or not at all) |
| 11 | Smoking history | A. Never B. Current smoker (smoked for ≥1 year) C. Former smoker (quit for ≥1 year) |
| 12 | Alcohol consumption history | A. Never B. Current drinker (≥1 time/week) C. Former drinker (abstinence ≥1 year) |
| 13 | Regular exercise (≥3 times/week, ≥30 min/session) | A. Yes B. No |

**Part II: Core Sleep Status Assessment**
*(Please answer based on your actual sleep over the past month)*

| **No.** | **Question** | **Options** |
| --- | --- | --- |
| 1 | What time do you usually go to bed at night? | A. Before 22:00 B. 22:00–23:00 C. 23:00–24:00 D. After 24:00 |
| 2 | How many hours of actual sleep do you usually get per night? | A. >7 hours B. 6–7 hours C. 5–6 hours D. <5 hours |
| 3 | How long does it usually take you to fall asleep after going to bed? | A. ≤15 min B. 16–30 min C. 31–60 min D. >60 min |
| 4 | Do you easily wake up during the night or wake up too early? | A. None B. <1 time/week C. 1–2 times/week D. ≥3 times/week |
| 5 | Does getting up to urinate at night disturb your sleep? | A. None B. <1 time/week C. 1–2 times/week D. ≥3 times/week |
| 6 | Do you experience breathing difficulties or severe snoring during sleep? | A. None B. <1 time/week C. 1–2 times/week D. ≥3 times/week |
| 7 | Does hypertension-related physical discomfort (e.g., headache, dizziness, limb numbness) affect your sleep? | A. None B. <1 time/week C. 1–2 times/week D. ≥3 times/week |
| 8 | Do you use medication to help you sleep? | A. None B. <1 time/week C. 1–2 times/week D. ≥3 times/week |
| 9 | Do you often feel drowsy during the day and have difficulty staying awake? | A. None B. <1 time/week C. 1–2 times/week D. ≥3 times/week |
| 10 | How would you rate your overall sleep quality over the past month? | A. Very good B. Good C. Poor D. Very poor |

**Part III: Supplementary Sleep-Related Factors**

| **No.** | **Question** | **Options** |
| --- | --- | --- |
| 1 | Is your living environment quiet? | A. Very quiet B. Relatively quiet C. Relatively noisy D. Very noisy |
| 2 | Do you use electronic devices (e.g., mobile phone, television) within 2 hours before bedtime? | A. Never B. Occasionally (<1 time/week) C. Often (1–2 times/week) D. Frequently (≥3 times/week) |
| 3 | Do you have the habit of drinking tea or coffee before bedtime? | A. No B. Occasionally C. Often |
| 4 | Do you have difficulty falling asleep due to emotional problems (e.g., anxiety, irritability, low mood)? | A. Never B. Occasionally C. Often D. Always |
